# Long-Term Protective Immune Responses Induced by rBCG-RBD/rRBD Heterologous Prime/Boost Immunization Strategy: Fusion of RBD-Wuhan with LTB Adjuvant Induces Cross-Reactivity with SARS-CoV-2 Variant Omicron

**DOI:** 10.3390/vaccines14020120

**Published:** 2026-01-27

**Authors:** Giana Carla Gaboardi, Monalisa Martins Trentini, Alex Issamu Kanno, Luana Moraes, Arthur Daniel Januzzi, Lennon Ramos Pereira, Greicy Brisa Malaquias Dias, Luciano Fernandes Huergo, Sergio C. Oliveira, André Bafica, Luciana Cezar de Cerqueira Leite

**Affiliations:** 1 Laboratório de Desenvolvimento de Vacinas, Instituto Butantan, São Paulo 05503-900, Brazil; giana_gaboardi@hotmail.com (G.C.G.); januzzi97@gmail.com (A.D.J.);; 2 Laboratory of Immunobiology, Department of Microbiology, Immunology and Parasitology, Federal University of Santa Catarina, Florianópolis 88040-900, Brazil; 3 Universidade Federal do Paraná, Setor Litoral, Matinhos 83260-000, Brazil; 4 Departamento de Imunologia, Instituto de Ciências Biomédicas, Universidade de São Paulo, São Paulo 05508-060, Brazil; scozeus1@gmail.com

**Keywords:** recombinant BCG, SARS-CoV-2, neutralizing antibodies, humoral and cellular immune responses, cross-protection against variants

## Abstract

**Background/Objectives**: SARS-CoV-2, the causative agent of COVID-19, has been responsible for more than seven million deaths worldwide since its emergence. The Bacillus Calmette–Guérin (BCG) vaccine, used for over 100 years to prevent tuberculosis, induces a Th1-prominent immune response that is important for protection against *Mycobacterium tuberculosis*, other mycobacteria, and intracellular pathogens. BCG has also been shown to induce innate immune memory and heterologous protection against non-related infections. Additionally, BCG has been used as a vector to express heterologous proteins, showing protective effects against various diseases, particularly respiratory viral infections, including SARS-CoV-2. In this report, we constructed two recombinant BCG strains as potential vaccine candidates based on the receptor-binding domain (RBD) of the Spike antigen: one expressing only the RBD protein (rBCG-RBD) and another expressing the RBD protein in fusion with the LTB (*Escherichia coli* Labile Toxin subunit B) adjuvant (rBCG-LTB-RBD). **Methods**: We evaluated the induction of SARS-CoV-2-specific humoral and cellular immune responses using these vaccine candidates in a prime–boost strategy with a booster dose using the rRBD protein (produced in cell culture) and the Alum adjuvant. Antisera were evaluated for neutralization of the Wuhan and Omicron SARS-CoV-2 pseudotyped virus. **Results**: Either immunization scheme (rBCG-RBD/rRBD or rBCG-LTB-RBD/rRBD) induced high IgG antibody titers, with antibody neutralization against a Wuhan SARS-CoV-2 pseudotyped virus after 10 weeks. The antibody levels induced by rBCG-RBD/rRBD were maintained for up to 9 months. Interestingly, only the sera from mice receiving the prime–boost with rBCG-LTB-RBD/rRBD showed cross-reactive neutralization against the Omicron SARS-CoV-2 pseudotyped virus. Immunization with rBCG-RBD or rBCG-LTB-RBD and a rRBD booster dose promoted the induction of specific CD4+ and CD8+ T cells producing Th1/Th2 cytokines (IL-4, TNF-α and IFN-γ). **Conclusions**: Our study highlights the potential of the prime–boost immunization strategy using rBCG-RBD/rRBD to induce long-term immunity and rBCG-LTB-RBD/rRBD to induce cross-protection against different variants, both of which could serve as promising vaccine candidates.

## 1. Introduction

The COVID-19 disease is caused by severe acute respiratory syndrome coronavirus 2 (SARS-CoV-2), which claimed the first victims in January 2020, with 767 million confirmed cases, including ~7 million deaths [1]. The disease is responsible for the largest pandemic of the century, mobilizing, in the last years, the collective efforts of international health institutions, government authorities, the pharmaceutical industry, and research groups around the world in the search for diagnostics, prevention, and treatment [1,2]. With the development of vaccines and their distribution to the population, the number of cases has drastically reduced, and since May 2023, it is no longer a global health emergency, as declared by the WHO. However, there were large differences in vaccination coverage between high- and low-income countries, which confers advantages for the virus to continue spreading and acquiring mutations. Therefore, there are peaks of infection from different variants. Among the reasons for the differences in immunization rates worldwide are the cost of the vaccines, logistics issues, and the need for special storage conditions of commercial vaccines [3]. The consequence of the insufficient vaccination rate in some regions is the emergence of several variants of concern (VOCs), with the current predominance of the Omicron variant strains. Although the available vaccines have reduced the number and severity of COVID-19 cases and deaths, the emergence of new VOCs contributes to the rapid dissemination of infection, while the unpredictable progression of the disease in some patients has overwhelmed hospitals [4].

The cellular receptor ACE2 is the target for SARS-CoV-2 entry into host cells. SARS-CoV-2 binds ACE2 with high affinity through the receptor-binding domain (RBD) of the Spike protein [5,6]. More than 90% of anti-Spike neutralizing antibodies (nAbs) in COVID-19 cases are specific against the RBD region [7,8], justifying the abundance of studies focused on RBD-containing vaccines [9,10,11,12]. Anti-spike nAbs exert an essential role in reducing viral load [13,14], and titers can remain stable for about 5 months post-infection, as reported in a study with around 30,000 individuals [15].

Most studies emphasize the role of nAbs in preventing virus entry but also indicate the importance of a SARS-CoV-2-specific T cell response to control the disease [16,17,18]. Furthermore, it has been shown that a protein vaccine inducing only cellular responses can induce protection against the virus in mice [19]. Despite its importance, nAbs have limited effects on the control of SARS-CoV-2 replication, and the clearance of the infection may depend on the development of an effective T cell response [18,20].

A strategy that could be explored in COVID vaccination is the mechanism described as innate immune memory, in which previous contact with a pathogen/antigen can induce protection against a non-related pathogen [21]. This effect has been observed following BCG vaccination [21,22,23]. Immunization of K18-hACE2 mice with a single dose of BCG:CoVac, a combination of BCG, Spike protein, and Alum, induced high titers of SARS-CoV-2 nAbs and almost completely abrogated disease after SARS-CoV-2 challenge, with minimal inflammation and no detectable virus in the lungs of infected animals. Boosting BCG:CoVac-primed mice with a heterologous vaccine (5 μg of Spike adjuvanted with Alum) further increased SARS-CoV-2-specific antibody responses [3]. In our previous study, mice were immunized with recombinant BCG expressing domains of the SARS-CoV-2 nucleocapsid and Spike proteins (rBCG-ChD6) followed by a boost with the homologous recombinant chimera protein, rChimera, with Alum as adjuvant. Prime–boost vaccination elicited high anti-Chimera total IgG and IgG2c Ab titers with neutralizing activity against SARS-CoV-2 Wuhan strain and induced IFN-γ and IL-6 production in spleen cells, with reduced viral load in the lungs after challenge [24]. These findings suggest the importance of evaluating this heterologous immunization strategy and reinforce the potential of vaccination based on recombinant BCG.

Certain bacterial toxins, such as the *E. coli* heat-labile toxin (LT), cholera toxin (CT), shiga-like toxin (SLT), and their derivatives, have proved to be potent immune adjuvants. Particularly, LT subunit B (LTB) has been extensively used in animals and even clinical trials, because it is generally deemed as being nontoxic since it has no enzymatic activity [25]. LTB has been used experimentally in numerous studies as a carrier for non-related antigens, with the ability to favor the induction of humoral responses and memory B lymphocytes [26]. Immunization with rLTB-RBD (produced in *E. coli* + Alum) showed significant IgG levels in mice [27]. Booster vaccination can be a potential alternative to the problem of waning immunity and viral diversification. Moreover, it has been suggested that achieving a higher neutralizing antibody titer with a booster dose is desirable to increase the breadth of neutralization [28,29].

In this context, we investigated the humoral and cellular immune responses induced in mice by two recombinant BCG strains expressing the RBD protein (rBCG-RBD) with or without fusion to the LTB adjuvant (rBCG-LTB-RBD), both followed by a boost with rRBD protein (produced in cell culture) in a heterologous prime–boost immunization strategy.

## 2. Materials and Methods

### 2.1. Bacterial Strains, Growth Conditions, and Vaccine Preparation

*Escherichia coli* DH5α was grown at 37 °C and 180 rpm in Luria–Bertani Broth (LB) (5 g/L yeast extract, 10 g/L tryptone, and 10 g/L NaCl; Sigma-Aldrich^®^, Saint Louis, MO, USA) and used for the vector propagation and cloning processes. *M. bovis* BCG strain Danish (ATCC #35733, Manassas, VA, USA) was used as control and to generate the recombinant BCG strains expressing the RBD and LTB-RBD sequences. wtBCG, rBCG-RBD, and rBCG-LTB-RBD were grown in Middlebrook 7H9 (MB7H9; Difco, Detroit, MI, USA) supplemented with 10% OADC (oleic acid–albumin–dextrose–catalase; BBL, Cockeysville, MD, USA), 0.5% glycerol (Sigma-Aldrich^®^), and 0.05% Tween 80 (Sigma-Aldrich^®^), containing 20 μg/mL kanamycin (Sigma-Aldrich^®^) or not. Transformed BCG was plated on solid Middlebrook 7H10 (MB7H10; Difco) supplemented with 0.5% glycerol and 10% OADC. Kanamycin (20 μg/mL) was added to the plates to select recombinant colonies. To prepare cell suspensions, the bacteria were incubated at 37 °C with 5% CO_2_ until cultures reached the exponential phase (OD600 = 0.6–0.8). Cells were harvested by centrifugation at low speed, washed twice with PBS, and resuspended in 10% Glycerol. wtBCG and rBCG preparations were maintained at −80 °C until used. Colony-forming units (CFUs) were determined 48 h after freezing. rRBD from Spike protein of SARS-CoV-2 (Wuhan variant) was expressed in EXPI293F™ cells (ThermoFisher Scientific, Waltham, MA, USA) and purified according to the manufacturer’s protocol.

### 2.2. Construction of the Mycobacterial Vectors for RBD or LTB-RBD Expression

The codon-optimized RBD gene expressing the (aa 319–541) RBD fragment (Wuhan) (GenScript, Piscataway, NJ, USA) was cloned into the mycobacterial vectors pLA71 and pLA73. Both vectors contain the *E. coli* and mycobacterial origins of replication, a kanamycin resistance gene (KanR), the upregulated *M. fortuitum* β-lactamase promoter, pBlaF*, its ATG initiation codon, and a multicloning site, which places the heterologous gene in fusion with either the β-lactamase signal sequence (ssBlam) or the whole β-lactamase encoding gene (Blam), in pLA71 or pLA73, respectively. Restriction sites for *Kpn*I and *Not*I enzymes were added before and after the gene, respectively, for insertion into the vectors. Alternatively, the codon-optimized LTB-RBD gene with a linker (GGGGSGGGGS) between them was also cloned into both pLA71 and pLA73 vectors. Thus, the gene encoding RBD is placed in fusion with ssBlam in pLA71-RBD, whereas the LTB-RBD sequence is in fusion with the whole Blam sequence in pLA73-LTB-RBD.

### 2.3. BCG Transformation and Expression of rBCG-RBD and rBCG-LTB-RBD

After cloning, competent BCG Danish was mixed with 500 ng of the resulting plasmids and electroporated using a Gene Pulser II device (BioRad, Hercules, CA, USA). Cells recovered in MB7H9 for 24 h and plated in supplemented MB7H10 agar plates with 20 µg/mL kanamycin until colony growth. BCG transformants resistant to kanamycin were grown in 5 mL of MB7H9-OADC-Kan at 37 °C and 5% CO_2_ and then used to inoculate 45 mL of MB7H9-OADC-Kan. The culture was incubated until the mid-log phase, when the cells were harvested by centrifugation, washed with PBS, resuspended in 1 mL PBS + 1% protease inhibitor cocktail (Sigma-Aldrich^®^), and lysed by sonication on ice for 5 min with an Ultrasonic Processor GE 100 (GE Healthcare). Protein concentration in the cell lysates was determined using a Bio-Rad DC Protein Assay (Bio-Rad), and bovine serum albumin (BSA) to make the standard curve. The protein extracts were separated by SDS-PAGE (Bio-Rad), transferred to a PVDF membrane (GE Healthcare, Chicago, IL, USA), and blocked with 5% skimmed milk powder at 4 °C overnight. The membrane was washed with PBS containing 0.05% Tween 20 (PBS-T) and incubated for 2 h with a polyclonal antibody anti-SARS-CoV-2-Spike-RBD region produced in rabbit, diluted 1:1.000 (Sigma-Aldrich^®^) to detect RBD expression. After membrane washing, a goat anti-rabbit IgG conjugated with peroxidase diluted 1:3.000 (Abcam, Boston, MA, USA) was incubated for 1 h. RBD in fusion with the FH8 tag of solubility and expressed in *E. coli* was used as positive control (C+). BCG Danish protein extract was used as negative control (C−). RBD detection was performed with a chemiluminescent ECL Prime Western Blotting System (GE Healthcare), and images were acquired with an Image Quant LAS4000 digital imaging system (GE Healthcare).

### 2.4. Animals and Immunization Schedules

The animal experiments were conducted ethically, following the Brazilian and international guidelines on animal experimentation, and approved by the Ethics Committee at Instituto Butantan (CEUAIB, São Paulo, SP, Brazil; protocol n° 2699240921). Female BALB/c mice at 6–8 weeks of age were bred and provided by the animal facility of Instituto Butantan, SP, Brazil, and housed in boxes in groups of 5 animals, with 12 h light/dark cycles, and a room temperature of 20–22 °C. To evaluate the immune response induced by the different prime–boost vaccination strategies, the animals were immunized subcutaneously (s.c, 100 µL) with 1 × 10^6^ CFU of either wtBCG, rBCG-RBD, or rBCG-LTB-RBD with a booster dose of rRBD (5 µg) combined with Alum (Alhydrogel, 50 μg) (Invivogen, San Diego, CA, USA) after 4 wks. A control group received only saline. Blood samples were collected every 2 or 4 wks after the prime, and the plasma was obtained by centrifugation at 3000 rpm for 10 min. Spleen and lungs were collected for the flow cytometry assays.

### 2.5. Antibody ELISA

Levels of anti-RBD IgG antibodies in mice were measured by ELISA. Then, 96-well plates (Corning, Corning, NY, USA) were coated with 5 µg/mL of rRBD, incubated overnight at 4 °C, and blocked with 10% skimmed milk powder. Sera were serially diluted from 1:25 to 1:51.200, added to the plates, and maintained for 1 h at 37 °C. Plates were washed and incubated for 1 h at 37 °C with goat anti-mouse IgG diluted at 1:10.000 (Southern Biotech, Birmingham, AL, USA) for IgG anti-RBD detection. After incubation and washing, the horseradish peroxidase-conjugated rabbit anti-goat IgG diluted at 1: 20.000 (Southern Biotech, cat n° 6160-05) was added and maintained for 1 h at 37 °C. Plates were revealed with TMB (BD OptEIATM, BD Biosciences, Franklin Lakes, NJ, USA) for 15 min at room temperature and protected from light. The reaction was stopped by adding a solution of H_2_SO_4_ 4N, and the absorbances were measured at 450 nm in a microplate reader (BioTek Instruments, Winooski, VT, USA). A purified mouse IgG was used to make the standard curve (Southern Biotech, cat. n° 0107-01). The cut-off value was calculated by the following formula: cut-off value = mean of absorbances of negative controls + 2SD (standard deviation). Sera titers were considered as the reciprocal of the dilution that presented a higher value than the cut-off. Positive control sera were also used for internal control of the reaction.

### 2.6. Pseudotyped Virus Sero-Neutralization

Plasmids and HEK293T/ACE2 cells for generation of the pseudotyped virus neutralization assay were kindly provided by Drs. Paul Bieniasz and Frauke Muecksch at Rockefeller University (New York, NY, USA). HEK293T cells were transfected using a Lipofectamine 3000 transfection reagent (Invitrogen, Carlsbad, CA, USA) mixed with the plasmids pNL4_3_dENV_NanoLuc and pSARS2_S_trunc, according to the manufacturer’s protocol. The transfected cells were incubated at 37 °C for 48 h. Cell supernatant was collected, centrifuged at 1300 rpm for 5 min at room temperature, and filtered with a 0.22 μm filter. SARS-CoV-2 pseudotyped virus was titered by serial dilutions of 10-fold up to 1 × 10^−6^, and the virus dilutions were incubated with 2.5 × 10^4^ HEK293T/ACE2 cells/well at 37 °C for 48 h. After that, Nano-Glo^®^ Luciferase Assay Reagent (Promega, Madison, WI, USA) was added to each well, mixed, and the cell lysate was transferred to a Lumitrac 200 microplate (Greiner Bio-One), which was incubated in darkness for 3 min at room temperature. The light produced was measured using a luminescence microplate reader (EnSpire™ Multimode Plate Reader (PerkinElmer Inc., Waltham, MA, USA) 0.5 s integration time) and expressed as RLUs (Relative Light Units). For the neutralization assay, the serum samples were inactivated (56 °C for 30 min), diluted in DMEM (Gibco, Waltham, MA, USA) according to previous standardization to a final dilution of 1:20 and added to 96-well plate to be incubated for 1 h at 37 °C with 50 μL/well of SARS-CoV-2 pseudotyped virus diluted to 1 × 10^6^ TCID50/mL, after that, 2.5 × 10^4^ HEK293T/ACE2 cells/100 μL was added to each well and the plate was incubated for 48 h at 37 °C. Sera of mice immunized with two doses of a commercial mRNA vaccine were included in the plate as positive control (C+), in a final dilution of 1:50. The luminescence was measured using Nano-Glo^®^ Luciferase Assay Reagent, as described above, and the results were expressed in RLUs. The infection inhibition rates were calculated according to the RLU values as follows: inhibition rate = [1 − (average RLU of sample average RLU of CC)/(average RLU of VC average RLU of CC)] × 100%, where CC—cell control wells; VC—virus control wells; PC—positive control; NC—negative control.

### 2.7. Flow Cytometry Assays

Spleen and lungs were collected from mice euthanized and placed in ice-cold RPMI-1640 (Sigma-Aldrich^®^). At first, lungs were incubated with collagenase III (0.7 mg/mL; Sigma-Aldrich^®^) for 90 min at 37 °C. To obtain single-cell suspensions, digested lungs and the spleens were macerated using syringe plungers and 70 µm cell strainers (BD Pharmingen™, San Diego, CA, USA), centrifuged for 15 min at 4 °C and 1300 rpm, and the supernatant discarded. In the pellet, the erythrocytes were lysed with 1 mL RBC lysis solution (150 mM NH_4_Cl, 10 mM KHCO_3_, pH 7.4), and then, the cells were washed once and resuspended in RPMI-1640 supplemented with 10% Fetal Bovine Serum (10% FBS). Cells were stained with Trypan Blue, and the counting was performed in a Neubauer chamber. Cell suspensions were adjusted to 10^6^ cells/well and plated in 96-well plates (Corning^®^). To assess RBD-specific cytokine induction by T cells, murine cells were stimulated with rRBD (10 μg/mL), anti-CD3 (1 µg/mL, clone:17A2, BD Pharmingen™), and anti-CD28 (1 µg/mL, clone: CD82.2, BD Pharmingen™) for 4 h at 37 °C and 5% CO_2_, and afterward, they were incubated with Protein Transport Inhibitor (BD GolgiStopTM, BD Biosciences, Franklin Lakes, NJ, USA) for 12 h. For the cell staining, the cells were incubated for 30 min with anti-CD4-PercP antibody (clone: RM4-5, BD Pharmingen™) and anti-CD8-BV421 antibody (clone: 53-6.7, BD Pharmingen™). After washing, cells were fixed and permeabilized using a Mouse Cytofix/Cytoperm Kit (BD) according to the manufacturer’s datasheet. The cells were further incubated for 30 min with anti-TNF-α-PE.Cy7 (clone: MP6-XT22, BD Pharmingen™), anti-IFN-γ-APC (clone: XMG1.2, BD Pharmingen™), and anti-IL-4-PE (clone: 11B11, BD Pharmingen™) for intracellular staining. Sample acquisition was performed on a BD FACSCanto II flow cytometer (BD Pharmingen™) and analyzed using FlowJoTM analysis software V10 (Treestar, Ashland, OR, USA). A total of 200.000 events were acquired for each sample. To calculate the total cell numbers for each cell subset, the absolute cell number previously determined by counting in a Neubauer chamber was multiplied by the percentage of that subset relative to the total lymphocyte gate.

### 2.8. Statistical Analysis

The data were presented as means ± standard deviation. Statistical analysis was performed in Graphpad Prism Software 9. The variances between the means of the experimental groups were compared and evaluated by Tukey’s multiple comparisons test, with individual variances computed for each comparison. To achieve normal distribution and variance homogeneity, antibody data were log-transformed before the statistical analysis. The difference between means was inferred as statistically significant when *p* < 0.05.

## 3. Results

### 3.1. Expression of RBD and LTB-RBD in Recombinant BCG

**Figure 1:**
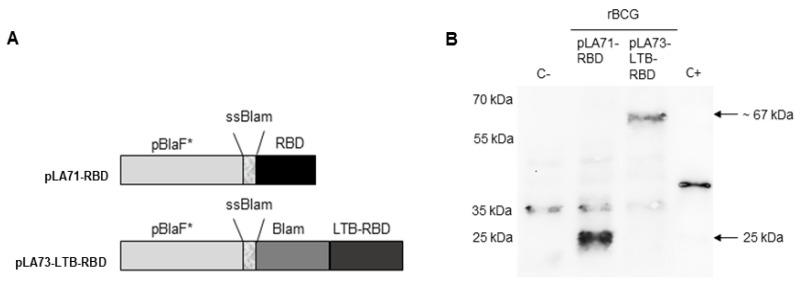
Expression of RBD and LTB-RBD in BCG. (**A**) Schematic representation of the expression cassettes of the vectors pLA71-RBD and pLA73-LTB-RBD. Both vectors contain origins of replication to *E. coli* and Mycobacterium, a kanamycin resistance gene (KanR), the pBlaF* promoter, and its ATG initiation codon. pLA71-RBD presents the RBD sequence in fusion with the β-lactamase signal sequence (ssBlam), generating ssRBD, while pLA73-LTB-RBD displays the LTB-RBD sequence in fusion with the whole β-lactamase encoding gene (ssBlam + Blam), generating Blam-LTB-RBD. (**B**) Expression of ssRBD and Blam-LTB-RBD in rBCG. Total cell extracts of wtBCG or rBCG (20 µg) were analyzed by Western blotting using an anti-RBD polyclonal antibody. Purified recombinant RBD in fusion with the FH8 tag for solubility and expressed in *E. coli* was used as positive control (C+), with approximately 45 kDa. Total protein extract of wtBCG was used as negative control (C−). Molecular weights are indicated on the left, and the expected molecular weight of ssRBD (25 kDa) and Blam-LTB-RBD (~67 kDa) are indicated by arrows. A non-specific band at 35 kDa is observed in all samples, including the negative control.

We used the mycobacterial expression vectors pLA71 and pLA73 to express either the RBD alone or in fusion with LTB (Figure 1A). BCG Danish was transformed with either pLA71-RBD or pLA73-LTB-RBD, and total cell extracts were analyzed by Western blot using a polyclonal anti-RBD antibody. In the rBCG-RBD construct, a band at 29 kDa was observed, which is the molecular mass of RBD (25 kDa) in fusion with ssBlam (4 kDa) (Figure 1B, Supplementary Figure S1). In the rBCG-LTB-RBD construct, a band at approximately 67 kDa was observed, comprising the fusion of Blam (~30 kDa), LTB (12 kDa), and RBD (25 kDa) (Figure 1B). A non-specific reaction occurred at ~30 kDa even in the negative control and possibly corresponds to a protein of wild-type BCG with cross-reaction to the polyclonal anti-RBD antibody. The constructions pLA71-LTB-RBD and pLA73-RBD did not show expression in BCG (Supplementary Figure S1).

### 3.2. Prime–Boost Schedule with rBCG-RBD/rRBD Induces Production of Neutralizing Anti-RBD Antibodies and a Cellular Response Against SARS-CoV-2

**Figure 2:**
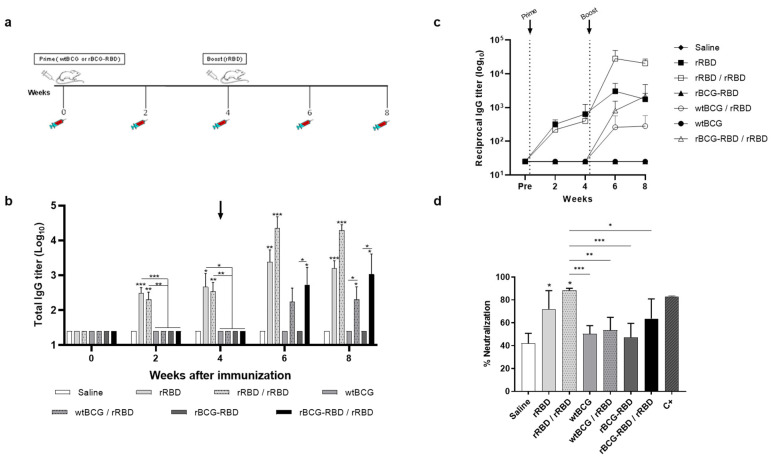
Prime–boost schedule with rBCG-RBD/rRBD induces production of anti-RBD antibodies against SARS-CoV-2 and neutralization of Wuhan SARS-CoV-2 pseudotyped viruses. (**a**) Experimental design for immunization by prime–boost schedule. BALB/c mice were immunized s.c. with wtBCG or rBCG-RBD (10^6^ CFU), receiving the boost dose of rRBD (5 µg) under the same conditions after 4 weeks (4 wk). Sera were collected on day 0 and every 2 wk until the final part of the experiment (8 wk). After the last blood collection, spleen and lungs were harvested to measure cellular immune responses and cytokine production. (**b**) Antibody response induced in sera of immunized mice was measured by anti-RBD total IgG antibodies by ELISA. (**c**) Antibody response induced in sera of immunized mice was measured by anti-RBD total IgG antibodies by ELISA. (**d**) Percent neutralization by sera of wtBCG- or rBCG-RBD-immunized mice with or without boost with rRBD, after 8 wk post-immunization (4 wk post-boost). Sera of mice immunized with two doses of a commercial mRNA vaccine were used as positive control (C+). Infectivity was quantified by measuring NanoLuc luciferase activity (RLU) following infection of HEK293T/ACE2 cells with the pseudotyped viruses. The sero-neutralization was obtained by the infection inhibition rates and calculated according to the RLU values. The black arrow indicates the boost administration. (*n* = 5 animals per group, * *p* values ≤ 0.05, ** *p* < 0.01, and *** *p* < 0.001 were considered statistically significant). Asterisks over the columns refer to the comparison with the saline group. Relevant statistical significance is indicated by the bars. Results are represented by the means ± SD and are representative of two independent experiments.

The humoral immune response induced by rBCG-RBD was investigated in a prime–boost schedule, where BALB/c mice were immunized with wtBCG or rBCG-RBD (10^6^ CFU), and after four weeks, they received a single dose of rRBD (produced in cell culture) (5 µg) with Alum (50 µg) as the adjuvant (Figure 2a). Groups of animals immunized only with the protein (rRBD—one or two doses) or only with wtBCG were used as positive and negative controls. Significant anti-RBD antibody levels were only detected two weeks after the boost (6 wk), whereas all the groups that received the rRBD boost had increased antibody levels (Figure 2b,c). Total anti-RBD IgG antibody titers in groups immunized with one or two doses of rRBD were significantly greater than in all the other groups. The groups immunized with wtBCG or rBCG-RBD followed by a boost with rRBD displayed increased anti-RBD antibody levels at 6 wks, and there was no increase in antibody production in any group at 8 wks.

The sero-neutralization assay was performed to assess the ability of antibodies to block the binding of Wuhan SARS-CoV-2 pseudotyped viruses to HEK293T/ACE2 cells at eight weeks post-prime (4 wk post-boost). Groups immunized with one or two doses of rRBD showed a significant percentage of neutralization, in agreement with the higher levels of anti-RBD antibody (Figure 2d). The groups immunized with wtBCG or rBCG-RBD with or without boost showed no significant production of nAbs at this timepoint, although the rBCG-RBD/rRDB group showed a tendency towards increased nAbs. Sera of mice immunized with two doses of a commercial mRNA vaccine were used only as a control of the method (C+) and not for comparative purposes, since the sera of the analyzed groups and the sera of the C+ were not tested at the same dilution.

**Figure 3:**
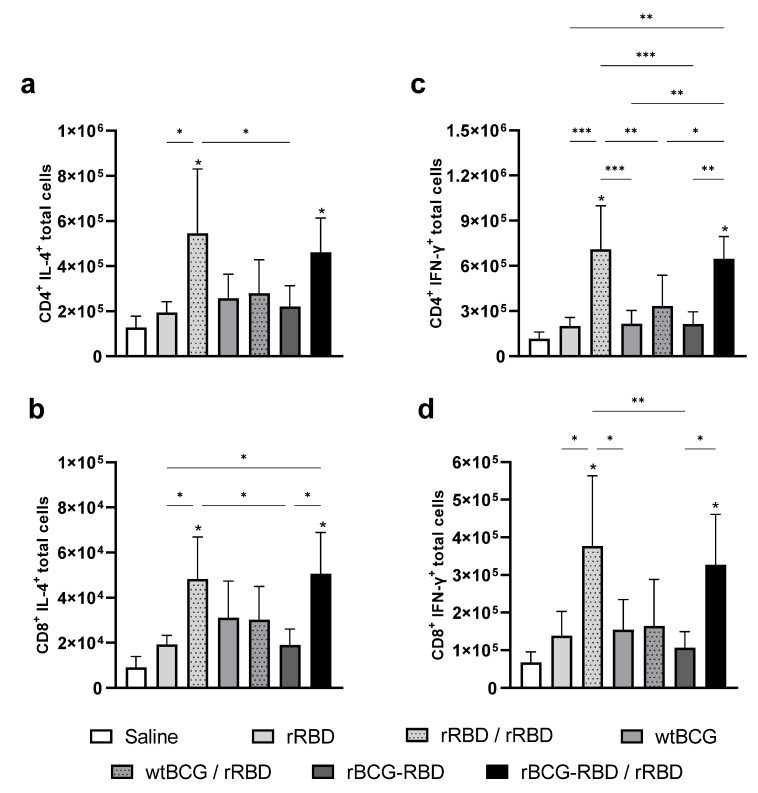
Cytokine production is induced by immunization with the prime–boost strategy using wtBCG or rBCG-RBD. Mice were immunized with wtBCG or rBCG-RBD (10^6^ CFU) and a boost with rRBD (5 µg) after 4 wk. Spleens of immunized mice were collected at 4 wk post-boost, and the cytokines produced by splenocytes were evaluated by Flow Cytometry. IL-4 production by CD4+ T cells (**a**) or CD8+ T cells (**b**) in rRBD-stimulated splenocytes; IFN-γ production by CD4+ T cells (**c**) or CD8+ T cells (**d**) in rRBD-stimulated splenocytes. The number of CD4+ and CD8+ T cells producing IL-4 and IFN-γ was analyzed using the Flow Cytometer FACS Canto II and FlowJo 8.7 software. (*n* = 5 animals per group, * *p* values ≤ 0.05, ** *p* < 0.01, and *** *p* < 0.001 were considered statistically significant). Asterisks over the columns refer to the comparison with saline group. Relevant statistical significance is indicated by the bars. Results are represented by the means ± SD and are representative of two independent experiments.

Since it is considered that the cellular immune response can contribute to protection in SARS-CoV-2 vaccines, we evaluated the Th1/Th2 cytokine profile in the spleens of immunized animals at 4 wk post-boost. Mice that received either prime–boost with rBCG-RBD/rRBD or two doses of rRBD showed increased production of CD4+ IL-4+ and CD8+ IL-4+ cells (Figure 3a and Figure 3b, respectively) at comparable levels. These groups also showed increased CD4+ IFN-γ+ and CD8+ IFN-γ+ (Figure 3c,d). Interestingly, prime with wtBCG and boost with rRBD showed no cytokine production. There was no significant increase in TNF-α production by either CD4+ or CD8+ spleen cells.

### 3.3. Prime–Boost Schedule with rBCG-RBD/rRBD Induces Long-Term Production of Anti-RBD Antibodies That Increases Following a Second Boost

**Figure 4:**
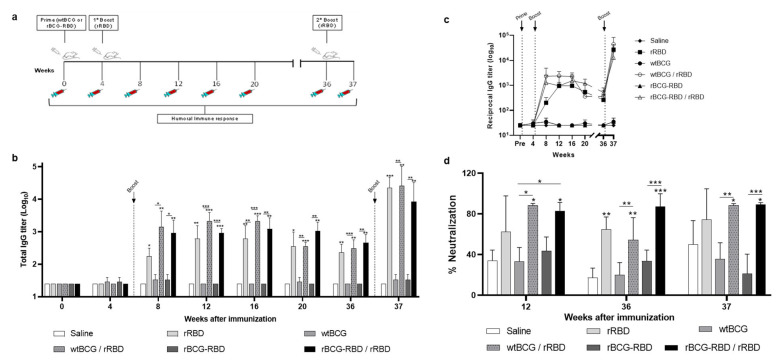
Prime–boost schedule with rBCG-RBD induces production of anti-RBD antibodies against SARS-CoV-2 up to 9 months and neutralization of Wuhan SARS-CoV-2 pseudotyped viruses. (**a**) Experimental design for immunization by prime–boost schedule. BALB/c mice were immunized s.c. with wtBCG or rBCG-RBD (10^6^ CFU), receiving the first boost dose of rRBD (5 µg) under the same conditions after 4 wk and a restimulation boost dose of rRBD (5 µg) at 36 wk. Sera were collected every 4 wk until 20 wk and then at 36 wk before the second booster and at 37 wk (post-second booster). (**b**) Antibody response induced in sera of immunized mice was measured by anti-RBD total IgG antibodies by ELISA. (**c**) Time course of anti-RBD total IgG antibody formation. (**d**) Percent neutralization by sera of wtBCG- or rBCG-RBD-immunized mice with or without boost with rRBD, after 12, 36, and 37 wk post-immunization. Sera of mice immunized with two doses of a commercial mRNA vaccine were used as positive control (C+). Infectivity was quantified by measuring NanoLuc luciferase activity (RLU) following infection of HEK293T/ACE2 cells with the pseudotyped viruses. The sero-neutralization was obtained by the infection inhibition rates and calculated according to the RLU values. The neutralization experiment was blinded. The first and second boosts are indicated by black arrows. * *p* values ≤ 0.05 were considered statistically significant. ** *p* < 0.01 and *** *p* < 0.001. Asterisks over the columns refer to the comparison with the saline group. Relevant statistical significance is indicated by the bars. Results are represented by the means ± SD.

Mice were initially immunized with wtBCG or rBCG-RBD (10^6^ CFU), receiving the boost with rRBD after 4 wk (Figure 4a). Anti-RBD antibody levels observed in the rBCG-RBD or wtBCG with a boost were maintained up to 36 wk (9 mo) (Figure 4b,c). The total anti-RBD IgG in the wtBCG/rRBD group showed a greater decline in antibody levels after 20 wk. A re-stimulation boost administered at 36 wk to the wtBCG/rRBD and rBCG-rRBD groups displayed a significant increase in the levels of antibodies detected at 37 weeks, 1 week post-re-stimulation (Figure 4b,c).

Sera were also evaluated for their ability to neutralize Wuhan and Omicron BA.1 SARS-CoV-2 pseudotyped viruses. Immunization with wtBCG or rBCG-RBD followed by a boost with rRBD generated sera with high neutralization (~80%) of the Wuhan variant pseudovirus at 12 wk, which persisted up to 36 wk (Figure 4d). Sera from the groups at 36 wk were analyzed by serial dilutions (1:20 up to 1: 1.280) to investigate their neutralization properties. The results showed that the rBCG-RBD/rRBD group displayed an LD50 of around 1:320–1:640 (Supplementary Figure S2). Following the re-stimulation boost, although total anti-RBD antibody levels were increased, neutralization levels in these groups were maintained after one week (Figure 4d). None of the groups produced antibodies that were able to neutralize the Omicron pseudovirus at any of the timepoints.

### 3.4. Prime–Boost Schedule with rBCG-LTB-RBD/rRBD Induces Neutralization of Wuhan and Omicron Pseudotyped Viruses

**Figure 5:**
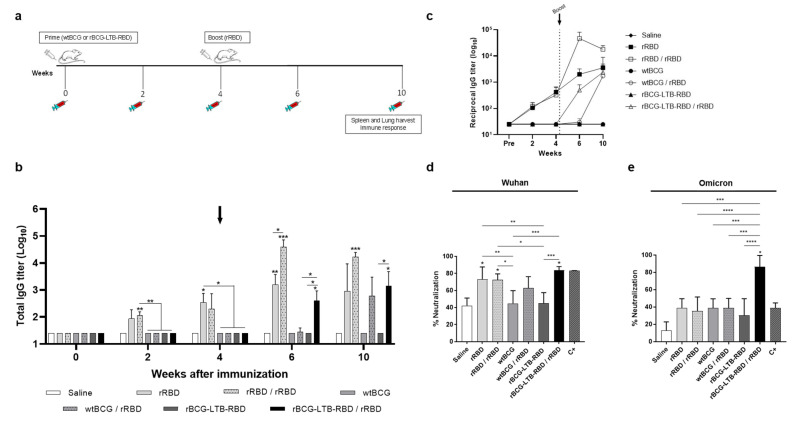
Prime–boost schedule with rBCG-LTB-RBD induces production of anti-RBD antibodies against SARS-CoV-2. (**a**) Experimental design for immunization by prime–boost schedule. BALB/c mice were immunized s.c. with wtBCG or rBCG-LTB-RBD (10^6^ CFU), receiving the boost dose of rRBD (5 µg) under the same conditions after 4 wk. Sera were collected on day 0 and at 2, 4, 6, and 10 weeks post-immunization. After the last blood collection, spleen and lungs were harvested to measure cellular immune response and cytokine production. (**b**) Antibody response induced in sera of immunized mice was measured by anti-RBD total IgG antibodies by ELISA. (**c**) Time course of anti-RBD total IgG antibody formation. Percent neutralization of Wuhan (**d**) and Omicron BA.1 (**e**) SARS-CoV-2 pseudotyped viruses by sera of wtBCG- or rBCG-LTB-RBD-immunized mice with or without boost with rRBD, after 10 wk post-immunization. Sera of mice immunized with two doses of a commercial mRNA vaccine were used as positive control (C+). Infectivity was quantified by measuring NanoLuc luciferase activity (RLU) following infection of HEK293T/ACE2 cells with the pseudotyped viruses. The sero-neutralization was obtained by the infection inhibition rates and calculated according to the RLU values. The boost is indicated by a black arrow. (*n* = 5 animals per group, * *p* values ≤ 0.05, ** *p* < 0.01, *** *p* < 0.001, and **** *p* < 0.0001 were considered statistically significant). Asterisks over the columns refer to the comparison with the saline group. Relevant statistical significance is indicated by the bars. Results are represented by the means ± SD and are representative of two independent experiments.

Since rBCG-RBD showed reasonable antibody levels and a cellular response, we attempted to increase antibody levels without decreasing the cellular response. We hypothesized that expressing the RBD in fusion with the LTB subunit (with known Th2-driving properties) could increase the humoral immune response. Mice primed with wtBCG or rBCG-LTB-RBD and receiving a booster dose with rRBD at 4 wk displayed significant IgG production at 6 wk (Figure 5b,c), increasing up to 10 wk (6 wk after the boost).

Regarding the production of nAbs, only the group receiving rBCG-LTB-RBD followed by a booster dose with rRBD exhibited significant neutralization titers, over 80%, for the Wuhan strain at 10 wk (Figure 5d). The groups receiving one or two doses of rRBD also displayed neutralization titers (~70%). When analyzing neutralization of the Omicron pseudovirus, only rBCG-LTB-RBD/rRBD showed a significant neutralization titer, over 80% (Figure 5e). As expected, sera from mice immunized with either one or two doses of rRBD showed no effect, nor did the positive control, where mice were immunized with the mRNA vaccine.

**Figure 6:**
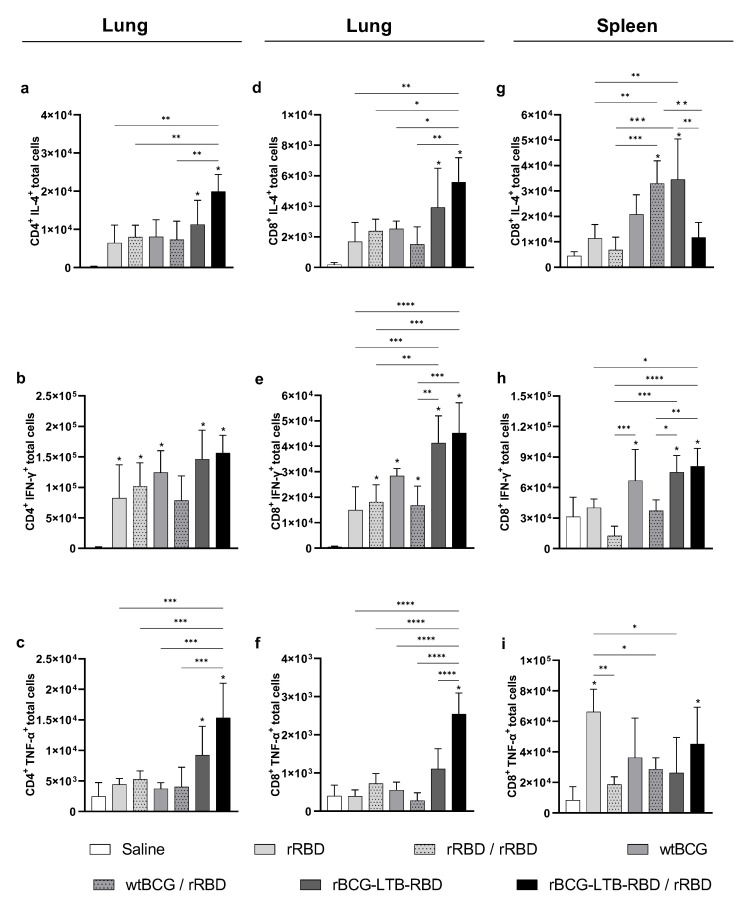
Th1 and Th2 cytokine production is induced by immunization with the prime–boost strategy using wtBCG or rBCG-LTB-RBD. Mice were immunized with wtBCG or rBCG-LTB-RBD (10^6^ CFU) and a boost with rRBD (5 µg) after 4 wks. Spleen and lungs of immunized mice were collected at 10 wks, and the cytokine production following rRBD stimulation was evaluated by Flow Cytometry. CD4+ T cells producing IL-4, IFN-γ, and TNF-α in the lungs (**a**,**c**,**e**); CD8+ T cells producing IL-4, IFN-γ, and TNF-α in the lungs (**b**,**d**,**f**); and the spleen (**g**–**i**). (*n* = 5 animals per group, * *p* values ≤ 0.05, ** *p* < 0.01, *** *p* < 0.001, and **** *p* < 0.0001, were considered statistically significant). Asterisks over the columns refer to the comparison with the saline group. Relevant statistical significance is indicated by the bars. Results are represented by the means ± SD and are representative of two independent experiments.

Evaluation of the Th1/Th2 and inflammatory responses revealed an increased presence of CD4+ and CD8+ T cells producing IL-4 in the lungs of the animals receiving rBCG-LTB-RBD with or without the rRBD boost (Figure 6a,b, gating strategy in Supplementary Figure S3). The levels of CD4+ IFN-γ+ were comparable in the different groups (Figure 6c). On the other hand, the levels of CD8+ IFN-γ+ were higher in the lungs of animals primed with rBCG-LTB-RBD, with or without the rRBD boost (Figure 6d). Both rBCG-LTB-RBD with or without rRBD boost induced significant production of CD4+ TNF-α+, and only rBCG-LTB-RBD/rRBD induced CD8+ TNF-α+ in the lungs of immunized animals (Figure 6e,f).

In the spleen, animals receiving rBCG-LTB-RBD/rRBD displayed a decrease in CD8+ T cells producing IL-4, whereas animals receiving only rBCG-LTB-RBD had increased levels (Figure 6g). No differences in CD4+ T cells expressing IL-4, IFN-γ, or TNF-α were detected. On the other hand, immunization with rBCG-LTB-RBD/rRBD showed increased levels of CD8+ T cells expressing IFN-γ and TNF-α (Figure 6h,i).

## 4. Discussion

Different strategies for development of SARS-CoV vaccines have been explored, and many were shown to be effective, generating nAbs and protection; several vaccines were made commercially available, and some still are. The vaccines used were highly efficient in reducing the burden of COVID-19 disease. However, the strategies employed showed short-term protection and low cross-reactivity against the new VOC. BCG has been shown to induce long-term protection and a broader immune response due to its heterologous protection. Therefore, we hypothesized that a recombinant BCG expressing SARS-CoV antigens could broaden and prolong the immune response.

We here developed rBCG strains expressing the Wuhan RBD gene, using mycobacterial vectors that contain the mutated ß-lactamase promoter from *Mycobacterium fortuitum* (BlamF*), previously shown to drive high levels of expression of different antigens [30]. We used pLA71, which expresses the antigen in fusion with ssBlamF*, and pLA73, which expresses the LTB-RBD in fusion with the whole β-lactamase molecule, Blam. Expression of the fusion proteins was shown by Western blot. It is interesting to note that we could not obtain expression using another vector that contains only the β-lactamase promoter, suggesting that initiation of expression through fusion with a mycobacterial protein can improve expression. On the other hand, we have previously obtained expression of a chimera of N and Spike epitopes without fusion to a mycobacterial protein. In this case, the vectors contained mutagenized promoters obtained through error-prone PCR using, as a template, the strong PL5 promoter, originally from mycobacteriophage L5 [31]. Therefore, it is not possible to ascertain whether the strength of the promoter or the fusion with mycobacterial proteins is more important for obtaining expression of the SARS-CoV antigens in BCG.

The non-specific immunomodulatory effects of the BCG vaccine, described as trained immunity potential, have been extensively recognized, including its role in enhancing the immune response and protection against heterologous infections [32,33]. In the context of COVID-19, several studies have demonstrated that BCG alone does not protect against viral infection but does modulate the clinical progression of the disease [21,34]. In our study, wtBCG vaccination followed by rRBD booster induced significantly higher levels of specific anti-RBD IgG at longer time periods and high levels of nAbs (key mediators of protection against SARS-CoV-2 infection). This enhanced humoral response may be attributed to the trained immunity induced by BCG, which potentially boosts the adaptive immune response to subsequent antigenic stimuli.

In addition to presenting trained immunity potential, the BCG vaccine has been widely investigated as a vector for expressing recombinant proteins, demonstrating protective effects against various infectious diseases [35,36,37]. Several studies have investigated the immune response induced by recombinant BCG vaccines against SARS-CoV-2 [24,34], employing different prime–boost immunization schedules. Studies using rBCG expressing the N protein of SARS-CoV-2 and boosted with the homologous protein [12,34] induced specific anti-N IgG antibodies. In our study, the rBCG-RBD vaccine, followed by a booster with the rRBD protein, induced high titers of specific anti-RBD IgG antibodies. In both studies the recombinant proteins were produced in eukaryotic cells. On the other hand, our previous studies using rBCG-ChD6 and a boost with the homologous rChimera protein, which was produced in *E. coli*, also displayed very high titers of anti-ChD6 antibodies. Both prime/boost schedules using rBCG with either eukaryotic or prokaryotic proteins displayed significant nAbs [24]. It is important to note that proteins produced in eukaryotic cells are glycosylated, which makes them highly immunogenic [38]. On the whole, these results indicate that glycosylation may not be essential for induction of protective immune responses against SARS-CoV-2 using an rBCG/rProtein prime–boost strategy. This can be an advantage since production of rBCG and recombinant proteins in *E. coli* usually involves lower costs.

In this study, we expressed the RBD in fusion with the LTB toxin derivative as an adjuvant, aiming to enhance the activation of dendritic cells and T and B lymphocytes, thereby boosting the antigen-specific antibody response, as previously described [39,40]. Mice immunized with rBCG-LTB-RBD/rRBD induced high titers of specific IgG antibodies, as well as a robust cellular immune response, predominantly Th1 (production of IFN-γ and TNF-α by CD4 and CD8 T lymphocytes). These results are in agreement with findings described by Maeda and colleagues, which showed that LTB co-administered with dengue antigens enhanced virus-neutralizing effects and modulated the epitope specificity of the antibodies generated [41]. These results suggest that, although the LTB fusion protein is expressed by BCG, it maintains its adjuvant potential, enhancing both cellular and humoral immune responses in viral vaccine formulations.

Neutralizing epitopes on the SARS-CoV-2 RBD domain, especially those overlapping the region associated with the ACE2 receptor-binding site, seem to be highly immunogenic and readily recognized by antibodies [20]. Here, we demonstrated that both rBCG-RBD and rBCG-LTB-RBD (with booster) induced high antibody titers with significant levels of nAbs. It has been demonstrated that the specificity, rather than the magnitude of the antibody response, is critical for virus neutralization [41]. Surprisingly, mice immunized with rBCG-LTB-RBD/rRBD exhibited high antibody neutralization against pseudotyped virus carrying both the SARS-CoV-2 Wuhan strain and the SARS-CoV-2 Omicron variant. The antibodies induced by the LTB-RBD protein expressed in BCG may display increased antibody/antigen interaction strength and/or different epitope recognition patterns [41,42,43].

Cellular immune responses are essential for the control and clearance of nearly all viral infections that cause disease in humans, and both cellular immunity and immune memory are pivotal to the success of all vaccines [17]. In this study, mice immunized with the rBCG-LTB-RBD vaccine (without the booster) induced a strong Th1-predominant cellular immune response (production of TNF-α and IFN-γ by CD4 and CD8 T lymphocytes). On the other hand, recombinant BCG expressing the SARS-CoV-2 N protein as a single dose was insufficient to elicit a robust cellular and humoral response [34]. Furthermore, both rBCG-RBD and rBCG-LTB-RBD with booster induced an enhanced cellular immune response (IL-4, TNF-α, and IFN-γ-producing CD4 and CD8 T cells) in the lungs of mice. A similar profile has been observed in mice immunized with recombinant BCG vaccines expressing the SARS-CoV-2 N protein [12] or an N-Spike chimera [24]. These findings are especially interesting, as the BCG vaccine is known to induce a predominantly Th1 profile with production of pro-inflammatory cytokines by CD4 T cells [44,45]. A mixed response, with both humoral and cellular (CD4 and CD8 T cells) immune responses, would be ideal for viral infections, such as SARS-CoV-2.

## 5. Conclusions

In summary, our study demonstrates (a) durable anti-RBD IgG induced by rBCG-RBD/rRBD, with Wuhan neutralization up to 9 months, and (b) Omicron BA.1 neutralization in the rBCG-LTB-RBD/rRBD group at 10 weeks. These results indicate that a prime–boost immunization strategy based on rBCG-LTB-RBD may elicit a long-term broad cross-reacting nAbs response against SARS-CoV-2, alongside a robust cellular immune response that may facilitate rapid infection control. This vaccination approach, using a prime with the rBCG platform in conjunction with a protein/alum boost, would be appropriate for human use and could be integrated into healthcare systems where BCG is already part of routine immunization. However, further investigation is warranted to evaluate the efficacy of this BCG-based vaccine platform against emerging VOCs of SARS-CoV-2.

## Data Availability

The original contributions presented in this study are included in the article/Supplementary Materials. Further inquiries can be directed to the corresponding author.

## Supplementary Materials

The following supporting information can be downloaded at: https://www.mdpi.com/article/10.3390/vaccines14020120/s1, Figure S1: Expression of RBD and LTB-RBD in BCG; Figure S2: Neutralizing activity was assessed by incubating mouse sera at serial two-fold dilutions (1:20 to 1:1280) for 1 h with a Wuhan SARS-CoV–based pseudovirus; Figure S3: Gating strategy to identify Th1/Th2 immune response in lungs and spleens.
