# Supplementary material for: Long-Term Protective Immune Responses Induced by rBCG-RBD/rRBD Heterologous Prime/Boost Immunization Strategy: Fusion of RBD-Wuhan with LTB Adjuvant Induces Cross-Reactivity with SARS-CoV-2 Variant Omicron
Source: Vaccines (Basel). 2026 Jan 27;14(2):120. doi: 10.3390/vaccines14020120 (PMC12944924; doi:10.3390/vaccines14020120)
Supplement: Supplementary file 1 [file vaccines-14-00120-s001.zip › vaccines-4061659-supplementary.pdf]

Supplementary Figures

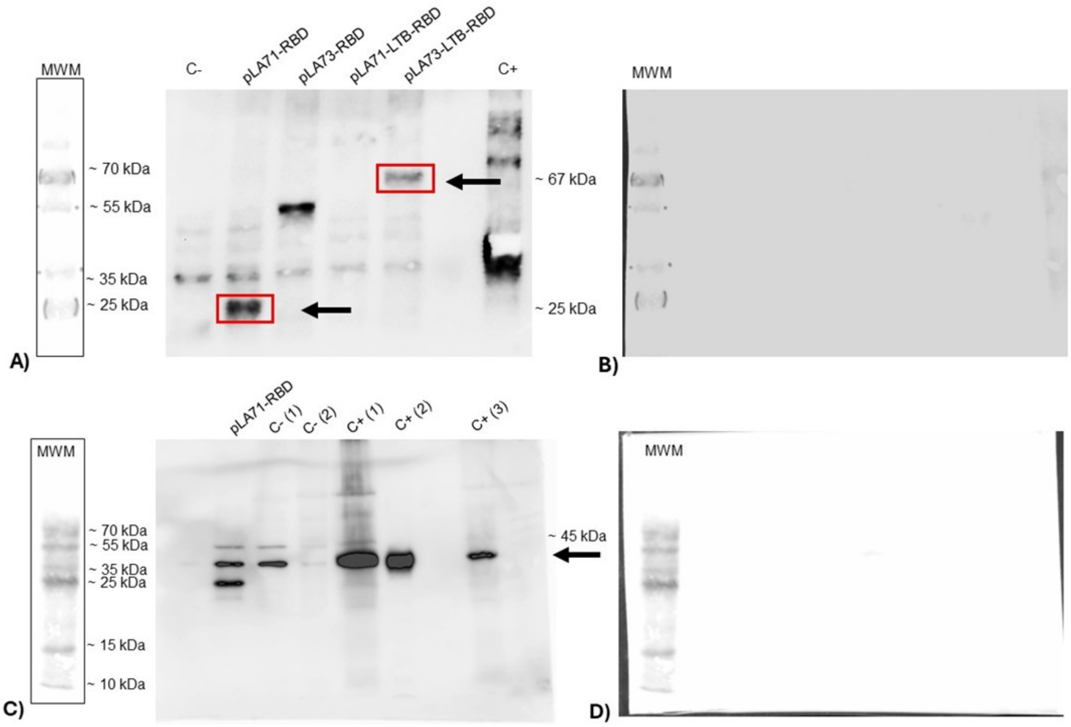

Supplementary Figure S1. Expression of RBD and LTB-RBD in BCG. (A) Total cell extracts of wtBCG or rBCG (20 μg) were analyzed by Western blotting using an anti-RBD polyclonal antibody (Anti-SARS-COV-2-Spike-RBD region, Sigma). Expression of ssRBD (25 kDa) and Blam-LTB-RBD (~67 kDa) in rBCG are indicated by arrows. The other samples (pLA73-RBD and pLA71-LTB-RBD) were not exploited in this study. Purified recombinant RBD in fusion with the FH8 tag of solubility and expressed in *E. coli* was used as positive control (C+), with approximately 45 kDa. Total protein extract of wtBCG was used as negative control (C-). Molecular weight marker (PageRuler Plus Prestained Protein Ladder - Thermo Scientific™) is indicated on the left. A non-specific band at 35 kDa is observed in all BCG samples, including the negative control. (B) Membrane picture prior to chemiluminescence revelation. Molecular weight markers are indicated in the first well on the left. (C) Western blot including rBCG-pLA71-RBD (20 ng), wtBCG (20 ng (C-1) and 4 ng (C-2)); positive control (rRBD) (C+) at different concentrations: C+ (1) (80 ng), C+ (2) (60 ng) and C+ (3) (20 ng). (D) Membrane picture prior to chemiluminescence revelation. Molecular weight markers are indicated in the first well on the left. The concentration of 20 ng (C+ (3) was considered ideal and used as positive control in Figure 1 of the paper.

a

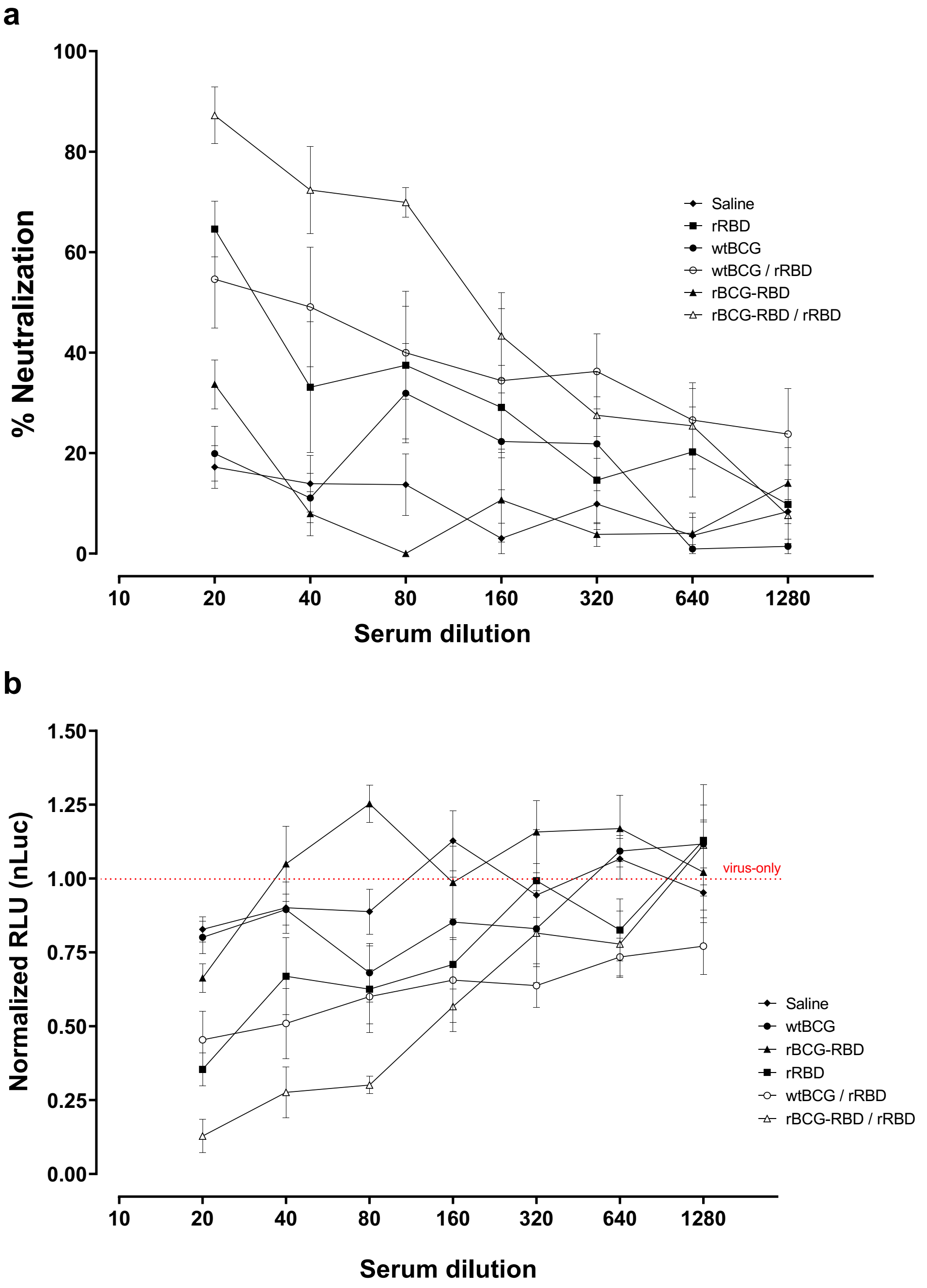

Supplementary Figure S2. Neutralizing activity was assessed by incubating mouse sera at serial two-fold dilutions (1:20 to 1:1280) for 1 h with a Wuhan SARS-Cov–based pseudovirus. Serum–pseudovirus mixtures were then used to infect HEK293T/ACE2 cells seeded in 96-well plates, and NanoLuc luciferase activity (RLU) was measured after 48 h. (a) Neutralization rates were calculated using the formula described in the Materials and Methods section, and results are presented as the percentage of neutralization. (b) RLU values shown on the Y-axis were normalized to the virus-only control (pseudovirus incubated in the absence of serum), which was set to a value of 1. Data is presented as the mean ± S.E.M and analyzed using GraphPad Prism software.

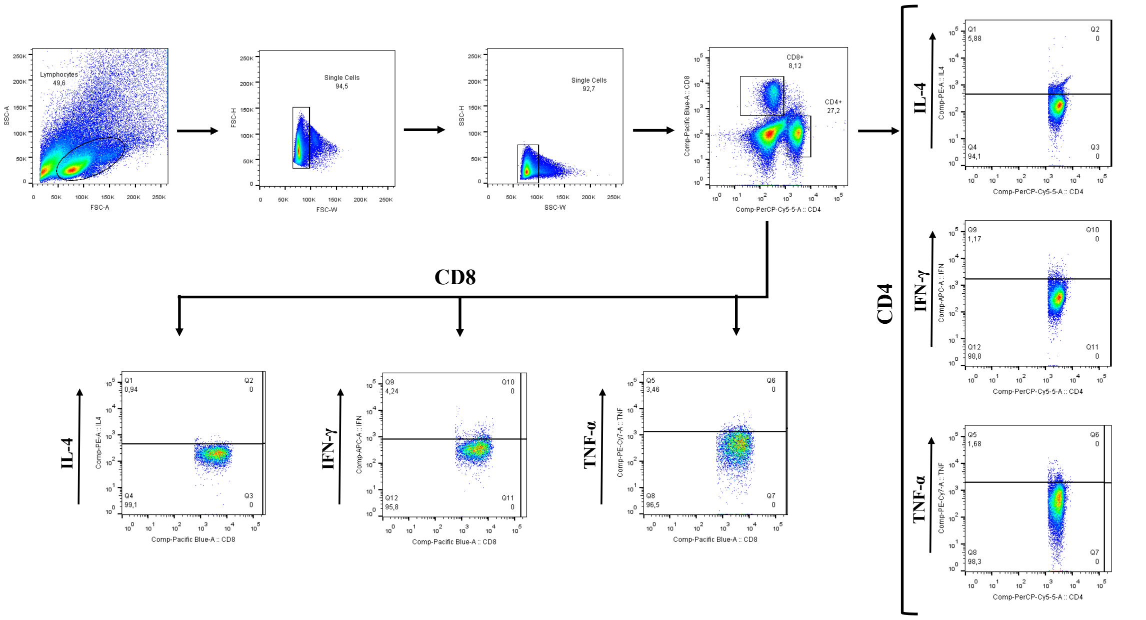

Supplementary Figure S3. Gating strategy to identify Th1/Th2 immune response in lungs and spleens. Gating strategy to identify lymphocytes and their cytokines. Spleen and lung cell suspensions were gated for lymphocytes by FSC (Forward scatter) and SSC (Side scatter). The lymphocytes were gated for single cells by FSC-H and FSC-W and then by SSC-H and SSC-W. The CD4+ and CD8+ cells were gated from single cells, and the different cytokines were quantified for both: CD8 + (CD8+ IL-4+, CD8+ IFN-γ+, and CD8+ TNF-α+) and CD4 + (CD4+ IL-4 +, CD4+ IFN-γ+, and CD4+ TNF-α+).
